# Progress in Psychiatry

**Published:** 1902-04-12

**Authors:** 


### Progress in Psychiatry.

(*Continued from page 9.*)

**Prognosis and Sequelæ of Melancholia.**—Simple or sub-acute melancholia, and acute melancholia in its milder grades are followed by recovery in the majority of patients when little or no hereditary predisposition is present. Such a melancholia lasts usually three to six months in the most favourable cases, but may last a year. At the end of the period the symptoms gradually subside, sleep becomes normal, the attacks of fear and gloom become less and less frequent, the facial expression becomes natural and brighter than during the illness, and interest and even sympathy is manifested for family and friends. Appetite is regained, the spirits rise, and cheerfulness begins to show itself. Notable mental improvement is accompanied by physical improvement, and both are gradual rather than sudden. The sense of individuality and personal identity becomes clear, and with advancing recovery the patient recognises that he has been ill and is getting well. "Although gradual improvement is the usual event, yet the changing symptoms of anxious melancholia and the severe phenomena of stupor are exposed to many variations during the period of convalescence, although they may terminate in complete recovery."[5] The patient may sometimes manifest a temporary exaltation of spirits before complete equilibrium is established. In other cases improvement may be taking place behind a mask as it were, till it shows itself at length in a marked improvement and goes on to rapid and complete recovery thereafter. Death does not occur in subacute melancholia or in the milder grades of acute melancholia, except as a result of intercurrent pneumonia or phthisis, the latter being a not infrequent complication. Most of the patients who recover regain their normal mental grade, but a few remain a little below their previous mental (intellectual and ethical) level. When delusions have not developed so far as to become organised in the brain during the course of the illness, they disappear as the brain recovers, and the same may be said of suicidal thoughts and promptings which have never developed to the extent of active suicidal attempts. Melancholia that develops in a brain invalided by hereditary taint or debilitated by serious acquired disease (influenza, severe anæmias, and chronic toxæmias) differs in some respects from melancholia developing in a brain previously healthy. In the former case there is often a mixture of morbid and normal elements, and the inexperienced observer may fail to diagnose the presence of melancholia since the patient will, with a show of reason, attempt to justify his or her conduct by attributing it to malign or sorrowful circumstances. These cases occur chiefly in women of dissatisfied lives, and who *ipso facto* (or from other causes) have developed morose and irritable dispositions. They are found to be incapable of work, quarrelsome, and malicious, and owing to their uncertain moods have perhaps for some time been reproached and then avoided by their relatives or friends. Any petty annoyance or fancied grievance will make them intensely melancholic, and in course of time (like the paranoiac)

they will manifest symptoms of melancholia which have been slowly accumulating and undergoing a certain degree of consolidation and logical cohesion. Such patients are especially prone to commit suicide. They may be rightly regarded as cases of *folie raisonnante* with a melancholic colouring. "Solitary-living old maids or bachelors become [thus] melancholic and morbidly suspicious, this being sometimes accompanied by auditory hallucinations,"[6] and such cases are by no means uncommon. As a rule they are not favourable as regards prospect of recovery. The melancholia which follows the exhaustion and stresses of the puerperium and of lactation is, next to hallucinatory delirium with mental confusion, the most frequent clinical form of the puerperal psychoses. Its average duration is 13 months (Hoppe), and from statistics collected from various authorities[7] it would appear that recovery may be looked for in about half of all such cases. The prognosis, however, gets progressively worse after second or third attacks. The prognosis may be regarded as bad (mentally) in those cases of melancholia which are characterised by fixed delusions—*e.g.*, delusions of damnation, of poisoning, of reproaches and accusations by "voices," and of having contracted loathsome diseases. The prognosis is also grave in cases of marked suicidal attempts, and when the patient's brain has been damaged by long-continued indulgence in alcohol. Such patients may have signs of polyneuritis. In the special form of melancholia marked by catatonic symptoms the prognosis is very grave for the mental life, as a permanent degree of mental enfeeblement and a tendency to chronicity and to dementia almost invariably succeeds. In the stuporous (anergic) form of melancholia, in the climacteric melancholia of women (associated with involution of the sexual life and its organs), and in the form described by Cotard under the name *délire de negation*, the prognosis is also unfavourable. Whenever the physical condition of a patient suffering from melancholia begins to improve without a corresponding return of mental integrity, the oncoming of dementia is to be feared. In women the approach of convalescence is indicated by a return of the menstrual functions which have suffered arrest during the course of the disease. The possibility of melancholia being a phase of circular insanity has to be borne in mind, as recovery is well-nigh hopeless in such cases. Senile mental decay may be characterised by periods of melancholia, variously intermingled with outbreaks of maniacal excitement and stupor. In some senile cases the melancholia is the predominant and most persistent symptom, combined, however, with a notable degree of amnesia and mental defect which examination may easily disclose. The prognosis is bad in cases of senile melancholia. In the examination of patients suffering from melancholia, careful search should be made for somatic disorders, and the thoracic and abdominal viscera (and in women the pelvic organs) should be examined with care. The temperature should be taken daily and repeatedly at the early stages of the disease so as to enable any intercurrent febrile complications to be recognised early. The termination of melancholia may be in complete recovery, in partial recovery, in death, in chronicity, or in secondary dementia. The majority of cases of subacute melancholia, and of acute melancholia in its milder grades, recover completely after a first attack. These recoveries probably amount to about 60 per cent., not including in this estimate climacteric and senile cases whose recovery rate is less favourable, and cases of post-puerperal and lactational melancholia whose recovery rate is also low. In a minority of cases recovery from a first attack of melancholia is partial; here, despite apparent recovery, careful investigation will reveal a "defect of the intellectual powers, a difficulty of entertaining complicated conceptions and judgments, which may easily escape the notice of the patient's friends."[8] The other terminations of a first attack of melancholia are chronicity, secondary dementia, or death in descending order of frequency. The course of subsidence of acute melancholia to chronicity is gradual and occupies years. The symptoms of simple misery and depression or of agitated moments (cries and gestures) are diminished in intensity though not necessarily in frequency. The actual distress and anguish experienced by the patient subsides, but its place is taken by a pseudo-suffering—a parrot-like repetition of groans and gestures which have from long repetition become stereotyped and automatic, but from which all true psychical significance as indicative of pain and suffering has long passed away. The face long moulded and scored by misery and pain still continues to exhibit the set expression of grief, as though the muscles of expression and the skin have become set and hardened like plaster of Paris, but the acuteness of pain and suffering has long subsided and the facial expression is only a relic, a mask that belies the present and only shows the past. Some of the former delusions, gestures, verbal expressions, and characteristic attitudes of walk or repose have also become automatic, and are repeated without meaning. This condition of chronicity may last for many years, the patient remaining thus enfeebled in some degree without actually sinking into dementia. Rarely, extraordinary delusions and transformation of personality may succeed. A small proportion of cases, however, after a first or second attack of melancholia, pass into a condition of torpor and stupidity which is indistinguishable from dementia. The mind becomes so enfeebled that scarcely any vestiges of intelligence remain. The head and shoulders droop, the jaw hangs relaxed, saliva dribbles from the half open mouth and chin, the patient is scarcely able to feed or dress himself, and has to be fed and attended to like an idiot child; he becomes entirely negligent of dress and person, and loses speech, or is only able to utter uncouth sounds and unintelligible words, or merely to repeat a few words or gestures automatically. He exhibits no mental activity, no initiative, no variety of acts, and probably experiences no emotions except those of the vegetative life—hunger, thirst, the sense of repletion, greed, and perhaps some degree of acquisitiveness. Many such patients are wet or dirty in their habits. Death from a first attack of melancholia is rare, and is due either to malnutrition and wasting (marasmus), intercurrent disease of the lungs (pneumonia, phthisis, bronchitis), gastro-intestinal disorders (including diarrhœa and colitis), or suicide.

[5] Kirchoff, loc. cit. [6] Clouston, in Scottish Med. and Surg. Jour., February, 1902. [7] Clouston, Hoppe, McLeod, Menzies, Lewis, Jelly, and Jones (vide Progress in Psychiatry, Oct. 26, 1901). [8] Petersen, Text-book of Mental Diseases, 1899.

